# Immune checkpoint inhibitor-associated thrombosis in patients with bladder and kidney cancer: a study of the Spanish Society of Medical Oncology (SEOM) thrombosis and cancer group

**DOI:** 10.1007/s12094-023-03171-z

**Published:** 2023-04-10

**Authors:** Manuel Sánchez Cánovas, David Fernández Garay, Evdochia Adoamnei, Esperanza Guirao García, Javier López Robles, Diego Cacho Lavin, Eva Martínez de Castro, Begoña Campos Balea, Alberto Garrido Fernández, Isaura Fernández Pérez, Asia Ferrández Arias, Noelia Suarez, Teresa Quintanar Verduguez, Miriam Lobo de Mena, Laura Rodríguez, David Gutierrez, Ana Manuela Martín Fernández de Soiginie, Silvia García Adrián, Ana Isabel Ferrer Pérez, María Jesús Delgado Heredia, Amelia Muñoz Lerma, Raquel Luque, Manuel Mazariegos Rubí, Ana Belen Rúperez Blanco, Ignacio García Escobar, Jaime Mendiola, Andrés Jesús Muñoz Martín

**Affiliations:** 1 Spanish Society of Medical Oncology (SEOM) Thrombosis and Cancer Group, Madrid, Spain; 2 Hematology and Medical Oncology Department, Hospital Universitario Morales Meseguer, Murcia, Spain; 3 Medical Oncology Department, Hospital Costa del Sol, Marbella, Spain; 4 Department of Nursing, Faculty of Nursing, University of Murcia, IMIB-Arrixaca, Murcia, Spain; 5 Medical Oncology Department, Hospital Universitario Marqués de Valdecilla, Instituto de Investigación IDIVAL, Santander, Spain; 6 Medical Oncology Department, Hospital Universitario Lucus Augusti, Lugo, Spain; 7 Medical Oncology Department, Hospital Álvaro Cunqueiro-Complejo Hospitalario Universitario de Vigo, Vigo, Spain; 8 Medical Oncology Department, Hospital General Universitario de Elche, Elche, Spain; 9 Medical Oncology Department, Hospital General Universitario de Valencia, Valencia, Spain; 10 Medical Oncology Department, Hospital de Fuenlabrada, Madrid, Spain; 11 Medical Oncology Department, Hospital Universitario de Móstoles, Madrid, Spain; 12 Medical Oncology Department, Hospital Obispo Polanco, Teruel, Spain; 13 Internal Medicine Department, Hospital Universitario de La Princesa, Madrid, Spain; 14 Medical Oncology Department, Hospital Virgen de las Nieves, Granada, Spain; 15 Medical Oncology Department, Hospital Universitario de Toledo, Toledo, Spain; 16 Social and Health Sciences, School of Medicine, University of Murcia, IMIB-Arrixaca, Cyber Epidemiology and Public Health (CIBERESP), Murcia, Spain; 17 Medical Oncology Department, Hospital Universitario Gregorio Marañón, Madrid, Spain

**Keywords:** Immune checkpoint inhibitors, Cancer-related thrombosis, Bladder cancer, Kidney cancer

## Abstract

**Purpose:**

Both venous and arterial thrombotic events (VTE/AT) can be associated with immune checkpoint inhibitors (ICI). However, there is a paucity of information *apropos* patients in routine clinical practice.

**Methods/patients:**

Retrospective, multicenter study promoted by the Thrombosis and Cancer Section of the Spanish Society of Medical Oncology (SEOM). Individuals with kidney or bladder cancer who initiated ICI between 01/01/2015 and 12/31/2020 were recruited. Minimum follow-up was 6 months (except in cases of demise). The primary objective was to calculate the incidence of ICI-associated VTE/AT and secondary objectives included to analyze their impact on survival and identify variables predictive of VTE/AT.

**Results:**

210 patients with kidney cancer were enrolled. The incidence of VTE/AT during follow-up (median 13 months) was 5.7%. Median overall survival (OS) was relatively lower among subjects with VTE/AT (16 months, 95% CI 0.01–34.2 vs. 27 months, 95% CI 22.6–31.4; *p* = 0.43). Multivariate analysis failed to reveal predictive variables for developing VTE/ AT.

197 patients with bladder were enrolled. There was a 9.1% incidence rate of VTE/AT during follow-up (median 8 months). Median OS was somewhat higher in patients with VTE/AT (28 months, 95% CI 18.4–37.6 vs 25 months, 95% CI 20.7–29.3; *p* = 0.821). Serum albumin levels < 3.5 g/dl were predictive of VTE/ AT (*p* < 0.05).

**Conclusions:**

There appears to be no association between developing VTE/AT and ICI use in patients with renal or bladder cancer. Serum albumin levels are a predictive factor in individuals with bladder cancer.

## Introduction

Immunotherapy (ICI) is increasingly widespread. As new studies are published, new indications are emerging. This means that in certain clinical scenarios, ICI is positioned ahead of chemotherapy or other antineoplastic therapy modalities. This increased use of ICI carries with it a higher prevalence of ICI-associated side effects.

Thrombotic events of venous (VTE) or arterial (AT) nature have been reported in the spectrum of immune-mediated toxicity [1]. This has led to research projects being conducted within the scope of what is known as “real world data”. Our group first approached patients with lung cancer or melanoma who received ICI at some point during their oncologic history [2].

The results of this study revealed that ICI-associated VTE/AT in these patients had a negative (and statistically significant) impact on survival. Furthermore, predictors were observed for this complication, such as the neutrophil/lymphocyte ratio or LDH levels when ICI therapy was initiated.

In the light of the findings, the decision was made to delve more deeply into this area. The second phase of this project was launched with the aim of analyzing ICI-associated VTE/ AT in people with bladder cancer or renal cancer. The reasons for choosing these tumors were the more widespread use of ICI to manage this patient profile, as well as their relevance in the TESEO registry [3], where bladder cancer has been found to be the seventh most thrombogenic cancer, while renal cancer ranks thirteenth. These data take on greater relevance when considering their prevalence in the Spanish population [4].

## Materials and methods

This study has been sponsored by the Thrombosis and Cancer Section of the Spanish Society of Medical Oncology (SEOM). It is a retrospective, multicenter study (14 centers). Data from patients with kidney cancer or bladder cancer who initiated ICI between 01/01/2015 and 31/12/2020 were collected. Selection was independent of tumor stage, type of ICI, or treatment intent. Participants had to have a minimum follow-up of 6 months (unless this was impossible due to patient demise).

Two independent cohorts were established, one consisting of cases of kidney cancer and the other, bladder. For both cohorts, the primary objective was to calculate the incidence of thrombosis associated with ICI. Two secondary objectives were defined. The first was to examine the impact of thrombosis on survival among subjects treated with ICI, while the second was to find predictor variables for the development of thrombosis VTE/TA.

Median and interquartile range (IQR) 25–75 were used to describe quantitative characteristics. Qualitative characteristics were reported by number (*n*) and percentage (%). Survival analysis was performed using the Kaplan–Meier estimator and log-rank test, calculating the median and 95% confidence intervals (CI) of survival times. In addition, analyses were performed with the “Landmark" method at 3, 6, and 9 months of follow-up from the time ICI therapy was initiated. To determine predictor variables, multivariate logistic regression models were performed to obtain Odds Ratios (OR) and 95% CI. Statistical significance was set at a p-value of 0.05 and the SPSS 25.0 statistical package (IBM Corporation, Armonk, NY, USA) was used.

The study was submitted to the Ethics Committee of each participating center and obtained the corresponding approval prior to its commencement. The processing, communication, and transfer of all personal data complied with the provisions of Organic Law 15/1999, dated December 13, 1999, regarding the protection of personal data and of Organic Law 3/2018, dated December 5, 2018, since it came into force.

## Results

### Kidney cancer

A total of 210 patients were recruited; baseline characteristics are displayed in Table 1. This cohort was predominantly male (74.8%) and had a median age of 63 years (IQR 56–69). Their functional status was good (93.2% with ECOG 0–1). Most had clear cell histology (57.7%) and disseminated oncological disease (98%, stage IV) when they started ICI.

**Table 1:** Baseline characteristics of the sample of kidney cancer patients (complete population and cohort with VTE/AT)

Parameter	Subparameter	Complete population (*n* = 210)	Cohort with VTE/AT (*n* = 12)
Gender	Male	74.8% (*n* = 157)	83.3% (*n* = 10)
	Female	25.2% (*n* = 53)	16.7% (*n* = 2)
BMI	< 18.5 kg/m^2^	2.0% (*n* = 5)	0% (*n* = 0)
	18.5–24.9 kg/m^2^	33.5% (*n* = 67)	41.7% (*n* = 5)
	25–29.9 kg/m^2^	42.5% (*n* = 85)	25% (*n* = 3)
	> 30 kg/m^2^	22.0% (*n* = 44)	33.3% (*n* = 4)
Smoking status	Never smoked	38.0% (*n* = 80)	33.3% (*n* = 4)
	Active smoker	21.0% (*n* = 44)	8.3% (*n* = 1)
	Ex-smoker	41.0% (*n* = 86)	58.3% (*n* = 7)
Medical history unrelated to the current kidney cancer	HTA	59.0% (*n* = 124)	58.3% (*n* = 7)
	DM	22.9% (*n* = 48)	33.3% (*n* = 4)
	DLP	43.8% (*n* = 92)	41.7% (*n* = 5)
	Thrombophilia	3.8% (*n* = 8)	8.3% (*n* = 1)
	Acute myocardial infarction	7.6% (*n* = 16)	0% (*n* = 0)
	Chronic CV disease	13.3% (*n* = 28)	16.7% (*n* = 2)
	Peripheral vascular disease	1.4% (*n* = 3)	0% (*n* = 0)
	COPD	3.8% (*n* = 8)	0% (*n* = 0)
	Autoimmune disease	4.3% (*n* = 9)	0% (*n* = 0)
	Liver disease	1% (*n* = 2)	0% (*n* = 0)
	CKD	11.9% (*n* = 25)	0% (*n* = 0)
	CVD	3.3% (*n* = 7)	0% (*n* = 0)
	Other previous malignancies	6.2% (*n* = 13)	8.3% (*n* = 1)
	VTE/AT (Diagnosed at least 30 days prior to the detection of kidney cancer)	5.3% (*n* = 9)	8.3% (*n* = 1)
	VTE/AT (Diagnosed between cancer diagnosis and ICI initiation)	16.7% (*n* = 35)	33.3% (*n* = 4)
	Concomitant hormonal therapy	1% (*n* = 2)	0% (*n* = 0)
	Concomitant EPO	0.5% (*n* = 1)	0% (*n* = 0)
	PICC or port-a-cath carrier	1.4% (*n* = 3)	0% (*n* = 0)
Tumor stage at ICI initiation	Stage III	2% (*n* = 4)	0% (*n* = 0)
	Stage IV	98% (*n* = 206)	100% (*n* = 12)
Histology	Clear cells	91.4% (*n* = 191)	100% (*n* = 12)
	Non clear cells	8.6% (*n* = 18)	0% (*n* = 0)
ECOG at start of ICI	0–1	93.2% (*n* = 193)	100% (*n* = 12)
	2–3	6.8% (*n* = 14)	0% (*n* = 0)
Treatment modality in which ICI was used	First-line metastatic disease	31% (*n* = 65)	50% (*n* = 6)
	Second-line metastatic disease	50% (*n* = 105)	50% (*n* = 6)
	Third or subsequent line of metastatic disease	17% (*n* = 36)	0% (*n* = 0)
	Neoadjuvant	2% (*n* = 4)	0% (*n* = 0)
Treatment regimen	Nivolumab in monotherapy	70.5% (*n* = 148)	50% (*n* = 6)
	Nivolumab plus ipilimumab	21.9% (*n* = 46)	33.4% (*n* = 4)
	Pembrolizumab plus targeted molecular therapy	3.3% (*n* = 7)	8.3% (*n* = 1)
	Others	4.3% (*n* = 9)	8.3% (*n* = 1)

*AT* arterial thrombosis, *CKD* chronic kidney disease, *COPD* chronic obstructive pulmonary disease, *CV* cardiovascular disease, *CVD* cerebrovascular disease, *DLP* dyslipidemia, *DM* diabetes mellitus, *EPO* erythropoietin, *HTA* arterial hypertension, *ICI* immune checkpoint inhibitors, *PICC* peripherally inserted central catheter, *VTE* venous thromboembolism

ICI was mainly used in the context of first- (31%) or second-line (50%) for advanced disease. Almost three quarters (70.5%) of the present cohort received nivolumab in monotherapy as an antineoplastic treatment modality.

Regarding thrombotic history, 5.3% of the subjects had a history of VTE/ AT. These events had been diagnosed at least 30 days prior to the detection of kidney cancer. In the interval between cancer diagnosis and date of ICI initiation, 16.7% of the cases had VTE/ AT.

The incidence of VTE/ AT associated with ICI during follow-up (median 13 months) was 5.7% (95% CI 3.30–9.72) (*n* = 12). Their baseline characteristics of VTE/AT episodes are depicted in Table 2. A median of 4.5 ICI cycles had been administered at the time of VTE/ AT diagnosis (IQR 3–11.8) and 41.66% of the patients in the cohort were receiving anticoagulant therapy (16.7% at prophylactic doses, 25% at therapeutic doses) at the time of event. As for VTE/ AT characteristics (Table 2), the most common form of thrombosis was pulmonary embolism (PE) (33.3%). Regarding arterial 8.3% of the arterial events affected the brain while the same percentage involved the heart.

**Table 2:** Characteristics of VTE/AT episodes in patients with kidney cancer

Parameter	Subparameter	*n* = 12
Type VTE/ AT	PE	33.3% (*n* = 4)
	DVT	8.3% (*n* = 1)
	Other forms of VTE: visceral, associated with catheter…	33.3% (*n* = 4)
	Cerebral stroke	8.3% (*n* = 1)
	Acute myocardial infarction	8.3% (*n* = 1)
	Other forms of AT	8.3% (*n* = 1)
Tumor reevaluation at diagnosis of VTE/AT	Complete response	0% (*n* = 0)
	Partial response	0% (*n* = 0)
	Stable disease	41.7% (*n* = 5)
	Unconfirmed progression	8.3% (*n* = 1)
	Confirmed progression	41.7% (*n* = 5)
	Not reevaluated	8.3% (*n* = 1)
VTE/AT presentation	Incidental	41.7% (*n* = 5)
	Symptomatic	58.3% (*n* = 7)
Setting of VTE/AT diagnosis	Outpatient	83.3% (*n* = 10)
	In-patient	16.7% (*n* = 2)
Setting of VTE/AT management	Outpatient	50% (*n* = 6)
	In-patient	50% (*n* = 6)

*AT* arterial thrombosis, *DVT* deep vein thrombosis, *PE* pulmonary embolism, *VTE* venous thromboembolism

In those cases in which patients’ cancer was reevaluated coinciding with the diagnosis of thrombosis (*n* = 11), oncological disease was found to be progressing in more than half of the participants (45.45% confirmed progressive disease [iCPD]; 9.09%, unconfirmed progressive disease [iUPD]).

More than half of the thromboses (58.3%) were symptomatic. Initial management was undertaken in hospital in 50% of the cohort, although most subjects (83.3%) were diagnosed in an outpatient setting. Following VTE/ AT, ICI was discontinued in 41.6% of the cohort. There were no instances of rethrombosis or bleeding events during follow-up post-VTE/ AT.

The multivariate analysis (Table 3) failed to reveal any statistically significant association between any of the variables analyzed and VTE/AT risk.

**Table 3:** Multivariate analysis to detect the relationship between clinical variables and development of VTE/AT in patients with kidney cancer and ICI

	Multivariate Analysis		
	HR	95% CI	*p* value
Liver metastases at initiation of ICI	0.53	0.60–4.72	0.572
Lung metastases at initiation of ICI	0.41	0.08–2.19	0.297
Bone metastases at initiation of ICI	0.56	0.08–3.71	0.548
ECOG at initiation of ICI (cutoff > 2)	1.13	0.28–4.63	0.858
Hemoglobin at initiation of ICI (cutoff < 10 g/dl)	0.83	0.52–1.33	0.449
Leukocytes at initiation ICI (cutoff < 10,000 cells/mm^3^)	0.28	0.02–5.32	0.401
Neutrophil/ lymphocyte ratio at initiation of ICI (cutoff < 3)	0.62	0.07–5.40	0.667
Platelet/ lymphocyte ratio at initiation of ICI (cutoff > 300)	4.16	0.46–37.7	0.205
Serum albumin levels (cutoff < 3.5 g/dl)	1.66	0.09–32.5	0.738
LDH levels (cutoff > 300 U/L)	1.13	0.22–5.97	0.879
Khorana score (cutoff > 1 point)	1.87	0.66–5.36	0.240

*CI* confidence interval, *HR* hazard ratio, *ICI* immune checkpoint inhibitors, *LDH* lactate dehydrogenase

**Fig. 1:**
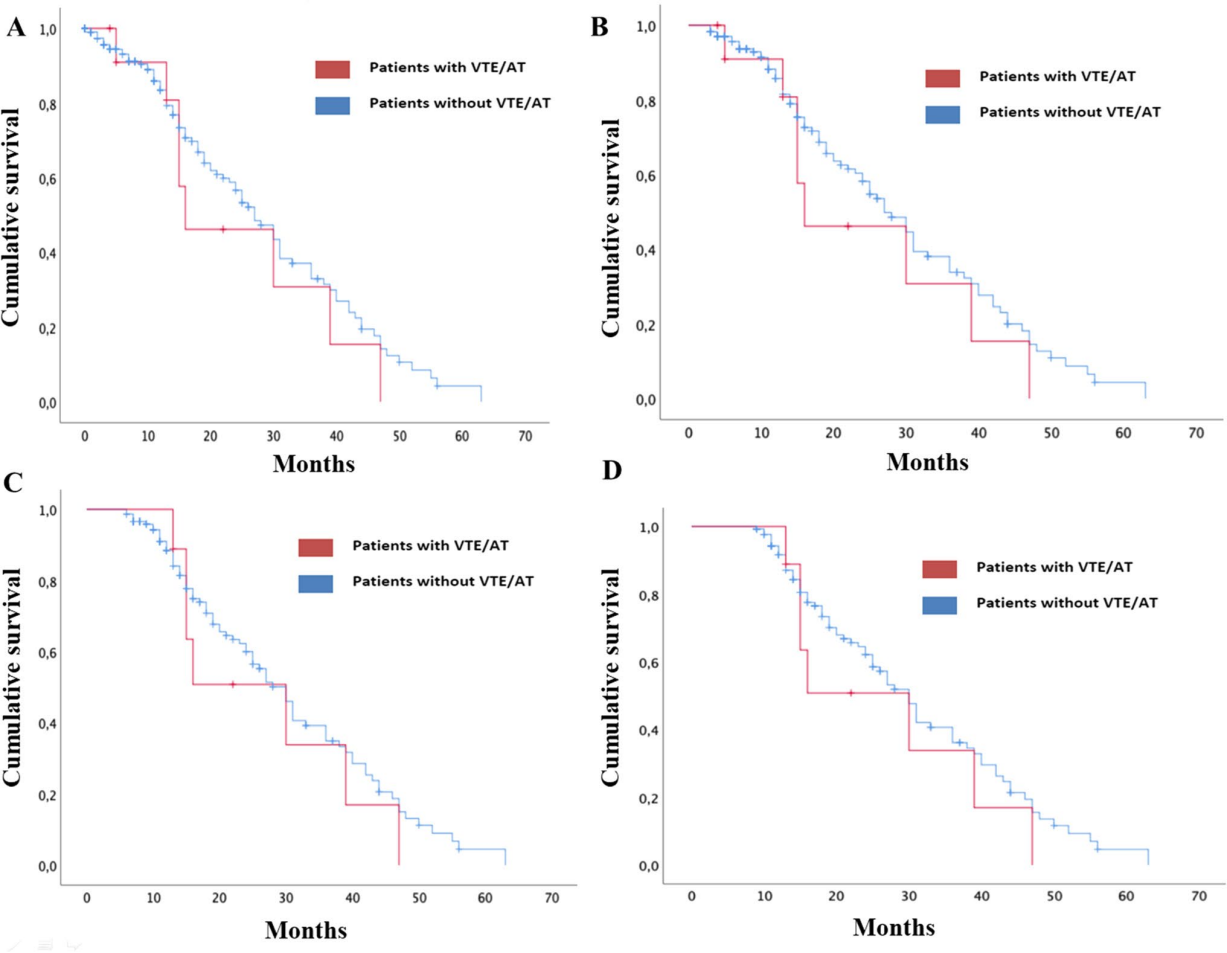
Survival analysis: **A** Kaplan–Meier curve comparing OS (since initiation ICI) of kidney cancer patients treated with ICI who developed VTE/ AT *versus* those who did not; **B** landmark analysis at 3 months after initiation ICI; **C** landmark analysis at 6 months after initiation ICI; **D** landmark analysis at 9 months after initiation ICI

Survival analysis (Fig. 1A) showed that median OS was relatively lower in the group with VTE/ AT (16 months, 95% CI 0.01–34.2) than in the group without VTE/AT (27 months, 95% CI 22.6–31.4); but differences were not statistically significant (*p* = 0.43).

Landmark analysis 3 months after starting ICI (Fig. 1B) yielded similar results with respect to the overall survival analysis (median OS in VTE/ AT group 16 months, 95% CI 0.01–34.2; non-VTE/ AT group, 27 months, 95% CI 22.7–31.3, *p* = 0.35).

Moreover, the same landmark analyses were performed at 6 months (Fig. 1C) and 9 months (Fig. 1D) following commencement of ICI were the same (median OS in VTE/AT group 30 months, 95% CI 11.9–48; non-VTE/AT group, 30 months 95% CI 26.5–33.5, *p* = 0.48 for the 6-months analysis and *p* = 0.39 for the 9-months analysis).

### Bladder cancer

A total of 197 patients were recruited. Their baseline characteristics can be found in Table 4. This cohort had a median age of 68 years (IQR 62–75), with a higher proportion of men to women (79.2% and 20.8%, respectively). Their functional status was good (91.8% ECOG 0–1). At the start of ICI, 99.5% of the subjects had stage IV disease. From a molecular perspective, PD-L1 had been determined in only 10.2% of the cohort with half of them (50%) being PD-L1 negative (< 1%). ICI was mainly used in second-line setting (65%). The most commonly used treatment regimens consisted of atezolizumab (86.3%) and pembrolizumab (11.2%) in monotherapy.

**Table 4:** Baseline characteristics of the sample of bladder cancer patients (complete population and cohort with VTE/AT)

Parameter	Subparameter	*n* = 197	*n* = 18
Gender	Male	79.2% (*n* = 156)	77.8% (*n* = 14)
	Female	20.8% (*n* = 41)	22.2% (*n* = 4)
BMI	< 18.5 kg/m2	2.6% (*n* = 5)	5.6% (*n* = 1)
	18.5–24.9 kg/m2	32.1% (*n* = 63)	22.2% (*n* = 4)
	25–29.9 kg/m2	40.3% (*n* = 79)	38.9% (*n* = 7)
	> 30 kg/m2	25% (*n* = 49)	33.3% (*n* = 6)
Smoking	Never smoked	25% (*n* = 49)	11.1% (*n* = 2)
	Active smoker	21.7% (*n* = 43)	33.3% (*n* = 6)
	Former smoker	53.3% (*n* = 105)	55.6% (*n* = 10)
Medical history unrelated to current bladder cancer	HTA	50.8% (*n* = 100)	33.3% (*n* = 6)
	DM	17.3% (*n* = 34)	16.7% (*n* = 3)
	DLP	37.1% (*n* = 73)	27.8% (*n* = 5)
	Thrombophilia	0.5% (*n* = 1)	5.6% (*n* = 1)
	Acute myocardial infarction	5.6% (*n* = 11)	0% (*n* = 0)
	Chronic CV disease	15.7% (*n* = 31)	11.1% (*n* = 2)
	Peripheral vascular disease	6.1% (*n* = 12)	16.7% (*n* = 3)
	COPD	13.2% (*n* = 26)	27.8% (*n* = 5)
	Autoimmune disease	8.1% (*n* = 16)	5.6% (*n* = 1)
	Liver disease	3.6% (*n* = 7)	0% (*n* = 0)
	CKD	9.7% (*n* = 19)	0% (*n* = 0)
	CVD	3% (*n* = 6)	0% (*n* = 0)
	Other previous malignancies	15.2% (*n* = 30)	11.1% (*n* = 2)
	VTE/AT (Diagnosed at least 30 days prior to the detection of bladder cancer)	4.1% (*n* = 8)	11.1% (*n* = 2)
	VTE/AT (Diagnosed between cancer diagnosis and ICI initiation)	13.2% (*n* = 26)	33.3% (*n* = 6)
	Concomitant hormonal therapy	1% (*n* = 2)	0% (*n* = 0)
	Concomitant EPO	2% (*n* = 4)	0% (*n* = 0)
	PICC or port-a-cath carrier	12.7% (*n* = 25)	16.7% (*n* = 3)
Tumor stage at ICI initiation	Stage I-III	0.5% (*n* = 1)	0% (*n* = 0)
	Stage IV	99.49% (*n* = 196)	100% (*n* = 18)
Histology	Urothelial	82.2% (*n* = 162)	88.9% (*n* = 16)
	Non-urothelial	17.8% (*n* = 35)	11.1% (*n* = 2)
PDL1	Undetermined/ Unknown	89.8% (*n* = 176)	88.9% (*n* = 16)
	< 1%	5.1% (*n* = 10)	5.6% (*n* = 1)
	1– 50%	3.1% (*n* = 6)	5.6% (*n* = 1)
	> 50%	2% (*n* = 4)	0% (*n* = 0)
ECOG at ICI initiation	0	28.9% (*n* = 57)	22.2% (*n* = 4)
	1	62.9% (*n* = 124)	72.2% (*n* = 13)
	2	8.1% (*n* = 16)	5.6% (*n* = 1)
Treatment modality in which ICI was used	First-line metastatic disease	25.9% (*n* = 51)	33.3% (*n* = 6)
	Second-line metastatic disease	65% (*n* = 128)	50% (*n* = 9)
	Third or subsequent line metastatic disease	0.5% (*n* = 1)	16.7% (*n* = 3)
	Neoadjuvant	17.5% (*n* = 51)	0% (*n* = 0)
Treatment regimen	Atezolizumab in monotherapy	86.3% (*n* = 170)	77.8% (*n* = 14)
	Pembrolizumab in monotherapy	11.2% (*n* = 22)	22.2% (*n* = 4)
	Others	2.5% (*n* = 5)	0% (*n* = 0)

*AT* arterial thrombosis, *CKD* chronic kidney disease, *COPD* chronic obstructive pulmonary disease, *CV* cardiovascular disease, *CVD* cerebrovascular disease, *DLP* dyslipidemia, *DM* diabetes mellitus, *EPO* erythropoietin, *HTA* arterial hypertension, *ICI* immune checkpoint inhibitors, *PICC* peripherally inserted central catheter, *VTE* venous thromboembolism

In terms of thrombotic history, 4.1% of patients had a positive history for VTE/ AT; these events had been diagnosed at least 30 days prior to the detection of bladder cancer. During the time between cancer diagnosis and start of ICI, 13.2% of the participants developed VTE/AT.

The incidence of ICI-associated VTE/ AT during follow-up (median 8 months) was 9.1% (95% CI 5.6–14.3) (*n* = 18). The baseline characteristics of VTE/ AT episodes are included in Table 4. A median of three ICI cycles had been administered at the time VTE/ AT were diagnosed (interquartile range 1–8). At that point, 50% of the subjects in the cohort were receiving anticoagulant therapy (22.2% at prophylactic doses, 27.7% at therapeutic doses). As for the characteristics of the VTE/ AT episodes (Table 5), DVT was the most common form of thrombosis (38.9%). As regards arterial events, we have only reported one event, an acute myocardial infarction.

**Table 5:** Characteristics of VTE/AT episodes in patients with bladder cancer

Parameter	Subparameter	*n* = 18
Type of VTE/ AT	DVT	38.9% (*n* = 7)
	PE	33.3% (*n* = 6)
	Other forms of VTE: visceral, associated with catheter…	22.2% (*n* = 4)
	Acute myocardial infarction	5.6% (*n* = 1)
Tumor reevaluation at diagnosis of VTE/ AT	Complete response	5.6% (*n* = 1)
	Partial response	5.6% (*n* = 1)
	Stable disease	5.6% (*n* = 1)
	Unconfirmed progression	5.6% (*n* = 1)
	Confirmed progression	50% (*n* = 9)
	Not reevaluated	27.8% (*n* = 5)
VTE/ AT presentation	Incidental	55.6% (*n* = 10)
	Symptomatic	44.4% (*n* = 8)
Setting of VTE/AT diagnosis	Outpatient	72.2% (*n* = 13)
	In-patient	27.8% (*n* = 5)
Setting of VTE/ AT management	Outpatient	38.9% (*n* = 7)
	In-patient	61.1% (*n* = 11)

*AT* arterial thrombosis, *DVT* deep vein thrombosis, *PE* pulmonary embolism, *VTE* venous thromboembolism

In those cases in which subjects’ cancer was reevaluated coinciding with the diagnosis of thrombosis (*n* = 13), oncological disease was found to be progressing in more than half of the participants (69.2% iCPD, 7.69% iUPD). More than half of the thromboses (55.6%) were incidental. Initial management was undertaken in hospital in 61.1% of the cohort, although most patients (72.2%) were diagnosed in an outpatient setting. After VTE/ AT, ICI was withdrawn in 50% of the cohort. As for post-VTE/ AT complications, no cases of rethrombosis were found during the follow-up period. Nevertheless, there were three bleeding episodes (16.6%), one of which was a major bleed.

Multivariate analysis (Table 6) revealed that one variable was seen to have a statistically significant association with VTE/ AT risk. This variable was serum albumin levels < 3.5 g/dl.

**Table 6:** Multivariate analysis to detect the relationship between clinical variables and development of VTE/AT in patients with bladder cancer and ICI

	Multivariate Analysis		
	HR	95% CI	*p* value
Liver metastases at initiation of ICI	0.87	0.10–7.22	0.894
Lung metastases at initiation of ICI	1.20	0.22–6.39	0.834
Bone metastases at initiation of ICI	1.17	0.14–9.95	0.884
ECOG at initiation of ICI (cutoff > 2)	0.99	0.06–16.47	0.990
Hemoglobin at initiation of ICI (cutoff < 10 g/dl)	1.0	0.64–1.7	0.80
Leukocytes at initiation ICI (cutoff < 10,000 cells/mm3)	0.49	0.05–5.38	0.562
Neutrophil/ lymphocyte ratio at initiation of ICI (cutoff < 3)	2.26	0.30–16.76	0.426
Platelet/ lymphocyte ratio at initiation of ICI (cutoff > 300)	0.71	0.10–5.10	0.730
Serum albumin levels (cutoff < 3.5 g/dl)	9.21	1.31–64.61	0.02
LDH levels (cutoff > 300 U/L)	0.61	0.11–3.3	0.570
Khorana score (cutoff > 1 point)	1.3	0.26–7.3	0.690

*CI* confidence interval, *HR* hazard ratio, *ICI* immune checkpoint inhibitors, *LDH* lactate dehydrogenase

**Fig. 2:**
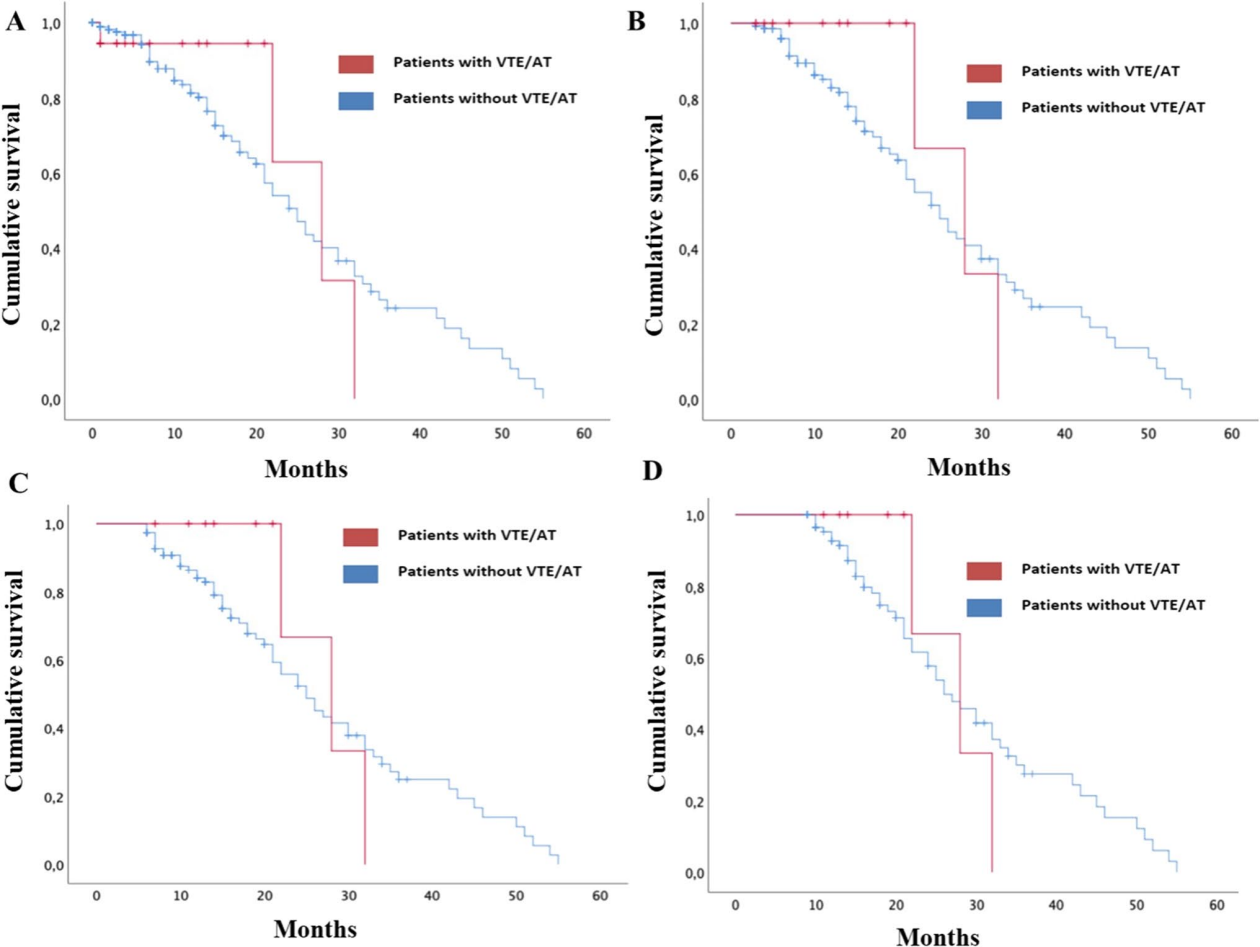
Survival analysis: **A** Kaplan–Meier curve comparing OS (since initiation ICI) of bladder cancer patients treated with ICI who developed VTE/AT *versus* those who did not; **B** landmark analysis at 3 months after initiation ICI; **C** landmark analysis at 6 months after initiation ICI; **D** landmark analysis at 9 months after initiation ICI

Survival analysis (Fig. 2A) revealed that median OS was somewhat higher in the group with VTE/ AT (28 months, 95% CI 18.4–37.6) than in the group without VTE/ AT (25 months, 95% CI 20.7–29.3); but the differences were not statistically significant (*p* = 0.82).

Landmark analysis at 3 months post ICI initiation (Fig. 2B) returned similar results with respect to the overall survival (median OS in VTE/ AT group 28 months, 95% CI 18.4–37.6; non-VTE/AT group 25 months, 95% CI 20.7—29.3, *p* = 0.571).

After 6-month landmark analysis (Fig. 2C), the trend was maintained (median OS in VTE/ AT group 28 months, 95% CI 18.4–37.6; non-VTE/AT group 25 months, 95% CI 20.2–29.8, *p* = 0.624). Finally, at 9 months, the results reflected no variations of interest (median OS in VTE/AT group 28 months, 95% CI 18.4–37.6; non-VTE/AT group 26 months, 95% CI 21.1–30.9, *p* = 0.932).

## Discussion

The relevance of thrombosis in a cancer patient’s history is incontrovertible. Data from the TESEO registry confirm that it constitutes, together with cancer itself, the second leading cause of death in this population [3]. This is reason enough to attempt to detect factors that may be related to the development, and even the evolution, of this complication.

The boom in ICI and the importance of VTE/AT led our research group to pursue the association between the two of them. After analyzing our sample, we proceeded to carry out an in-depth review of the literature for the purpose of finding series that might be comparable to ours. Nevertheless, to the best of our knowledge, this is the first study to analyze independently cohorts consisting exclusively of individuals with bladder or kidney cancer. This does not mean, however, that there are no data available concerning this patient profile from studies involving a more heterogeneous oncologic population.

The challenge with all these series is that subjects with renal and bladder cancer account for a small percentage of the study samples, owing to the low prevalence of these types of tumors in the population [4–6]. For instance, the data reported in the series by Moik et al. [7] and Gutierrez-Sainz et al. [8], the population with renal cancer constitutes 11% (*n* = 74) and 11.8% (*n* = 27), respectively, while the percentages of subjects with bladder cancer are 4.9% (*n* = 33) and 7% (*n* = 16). Other series by Gong et al. [9] or Kewan et al. [10] group patients with genitourinary tumors in the same category, reporting 6.3% (*n* = 174) and 23.2% (*n* = 128), respectively.

Taking these series as a reference, we find the absolute number of patients included in each cohort to be compelling. It is at this point that the data provided by the present work, in terms of renal and bladder cancer, may be of greater scientific relevance, as much as 210 patients were included in the former group and 197 in the latter. Add to this the multicenter nature of this study, and the geographical variability (within our country) does not represent any bias or limitation in terms of the conclusions drawn.

With these limitations in consideration, it is understandable that our series' survival results cannot be compared with any other because, as far as we are aware, ours is the first to perform these analyses in population groups made up exclusively of subjects with renal and bladder cancer. Hence, it is impossible to know whether those results not achieving statistical significance in terms of survival would be replicated with patients from other populations. Nevertheless, this does not prevent us from finding it intriguing that the impact of ICI-associated VTE/AT on survival has not been established, while this objective was achieved in the lung cancer and melanoma series we studied following the same methodology [2].

One of the factors that might justify this could be the oncologic disease itself. In our previous study [2], lung cancer/ melanoma patients received ICI (mainly) in a first-line metastatic disease setting. In contrast, in this second study, approximately 65% of the sample received ICI as second (or subsequent) line for disseminated disease. Therefore, this is a population that, by definition, has a worse prognosis and the effect of VTE/ AT may be diluted by that of the cancer itself.

One should also bear in mind that this analysis does not include a significant number of patients belonging to groups in which the use of ICI has recently been approved and extended [11], such as those receiving avelumab as maintenance therapy for first-line bladder cancer with metastatic disease.

In general, these limitations could be overcome if different research groups may conduct survival analyses of cohorts comprising only a single type of tumor pathology. Thus, the series would be comparable and it would be easier to draw conclusions. Even so, such series would necessarily have to include a sufficient number of cases to ensure that the analysis would have adequate statistical power.

In addition to survival, another relevant aspect to be discussed is the detection of factors that can predict the development of ICI-associated VT/TA. As with the previously mentioned studies [7–10], the fact that the analysis was not performed on an exclusive patient population with renal or bladder cancer means that the data are not comparable. Similarly, another relevant aspect is that not all the series analyze the same variables in the logistic regression.

Even so, there are aspects that deserve attention; for instance, ECOG performance status when initiating treatment with ICI. The series by Moik et al. [7] do not show that this parameter can predict the development of ICI-associated VTE/AT, which coincides with our series. However, the one analyzed by Kewan et al. [10] does confirm this parameter as a predictor of ICI-associated VTE/AT.

On the other hand, we would like to highlight our findings regarding albumin in bladder cancer patients receiving ICI. The lower the albumin levels, the lower the muscle mass and therefore the greater the probability of anorexia-cachexia syndrome. With an established anorexia-cachexia syndrome, the functional situation should be worse and therefore, the risk of VTE/TA higher, although the latter is not consistent with the absence of statistical significance for the ECOG variable for predicting ICI-associated VTE/TA. Likewise, we have been unable to find an explanation as to why hypoalbuminemia is a predictor of VTE/AT in patients with bladder cancer and not in those with renal cancer.

As with the survival analysis, we believe that it would be worthwhile for research groups to analyze cohorts solely comprised of a single tumor type so as to be more precise in defining and comparing predictive factors for ICI-associated VTE/AT. Likewise, if a protocol were established, that would allow us to define a set of common variables to perform multivariate logistic regression, and it would be easier to compare series and obtain meaningful information.

Another aspect that can influence the comparability of the results is geographic distribution. For example, Chiang et al. [12] carried out an analysis exclusively in an Asian population with different types of tumors treated with ICI. While logistic regression reflected that there were several predictors of ICI-associated VTE/AT (age, metastatic disease, hypertension, platelet/lymphocyte ratio) the results cannot be considered fully generalizable to our series as ethnicities are different.

Regardless of the hypotheses that seek to explain the results we have obtained in our series and the limitations when comparing with other findings, we believe further research in this area to be necessary, as there is increasing evidence to suggest that there may be an association between the use of ICI and VTE/AT. Alghamdi et al. [13] collected clinical cases of oncology patients receiving ICI. Of their sample, 14.9% had renal cancer and 7.45% had urothelial carcinoma. The data examined suggested that there may be an association between the use of ICI and VTE/AT.

Despite the strengths of the study, one must not overlook the limitations of this project. The most important of these is its retrospective nature. Likewise, it must be remembered that the indications and availability of ICI continue to grow as time goes by. This implies that should the analysis be repeated in the future, with a larger sample and even ambispective or prospective data, results could change. We therefore do not consider this study to be the end of the chapter on clinical research in the field of ICI-associated VTE/AT.

With all the data that have been exposed throughout the article, and after an exhaustive bibliographical review, there is no solid scientific evidence that allows establishing a definitive association between VTE/AT and ICI. More research is required in this field. Not everything can be limited to data from clinical trials, the field of real-world data is essential. This type of research, focused on each type of tumor and on the different types of ICI, could lead to systematic reviews and meta-analyses that provide conclusions in this area.

## Conclusions

Based on the data from our series, there appears to be no association between the development of VTE/ AT and the use of ICI in subjects with renal or bladder cancer. Serum albumin levels are a predictive factor for this complication in the subgroup of patients with bladder cancer.

## Data Availability

The authors declare the availability of data analyzed in this study.
